# Woodlands and woody debris: Understanding structure and composition to inform restoration

**DOI:** 10.1371/journal.pone.0224258

**Published:** 2020-03-05

**Authors:** Adrian D. Manning, Ross B. Cunningham, David Tongway, David B. Lindenmayer

**Affiliations:** The Fenner School of Environment and Society, The Australian National University, Canberra, ACT, Australia; Chinese Academy of Forestry, CHINA

## Abstract

Simplification of stand structure of forests and woodlands through human-induced modification is a serious threat to biodiversity. Restoring lost habitat complexity and heterogeneity, such as woody debris, requires an understanding of the relationships between different elements that contribute to stand structure. In this study, we examine the structure and composition of a critically endangered box-gum grassy woodland in south-eastern Australia and relationships with woody debris loads. We found that: (1) despite modification by humans and differing susceptibility to dieback, the two dominant tree species, Blakeley’s red gum, *Eucalyptus blakelyi* and yellow box, *E*. *melliodora*, occurred in similar proportions irrespective of vegetation density; (2) *E*. *blakelyi* had the largest number of stems and basal area, but while *E*. *melliodora* had fewer stems, it had a similar basal area to *E*. *blakelyi*. *E*. *melliodora* also showed fewer signs of dieback than *E*. *blakelyi* with between 40–50% trees in good condition compared to 2% for the latter species; (3) woody debris loads were low compared to other studies in woodland, but there were levels of heterogeneity indicating ‘natural’ accumulation was occurring; (4) tree basal area and woody debris loads had a 1:1 relationship across all sites and vegetation densities. Overall, our study indicated that ecosystem recovery was taking place (i.e. with many young trees), but there were fewer large trees that are known to supply most woody debris. Our findings highlight the slow accumulation of this critical resource because the volumes were lower than expected. Based on our results, we recommend: (1) aiming for approximately a 50:50 ratio of yellow box to Blakely's red gum basal area in woodland restoration projects; (2) to accelerate the recovery of woodland structure, addition of woody debris should be added at a minimum ratio of 1:1 to standing basal area (i.e. a basal area of 5.99 m^2^ requires a minimum volume of 3.11 m^3^) (3) managing for both volume and heterogeneity of woody debris loads; (4) preserving large diameter trees to harness proportionally higher woody debris and litter inputs.

## Introduction

The structure and composition of vegetation is critically important for maintaining biodiversity, ecological processes and ecosystem services [1, 2]. For example, vegetation structure and plant composition influences habitat for birds [3–6], reptiles [7, 8] [9], mammals [10] and invertebrates [11]. Structure and composition also affects ecosystem processes, such as inputs of woody debris [1], the extent, intensity and return rate of fire [12], the behaviour and distribution patterns of fauna through varying the risk of predation [13–15].

Studying the structure and composition of a woodland provides information about its past, and its potential future [16, 17]. Manipulation of that structure and composition through management is a way of influencing the future of a forest or woodland, and its value for biodiversity, ecological processes, and ecosystem services.

The maintenance, restoration and spatial expansion of woodlands through active management is particularly important where an ecosystem type is highly modified and greatly reduced in extent. A good example is the box-gum grassy woodlands in south eastern Australia, which occur in an internationally recognised endangered ecoregion [18]. Box-gum grassy woodland is an ecological community dominated by mixtures of yellow box (*Eucalyptus melliodora*) and Blakely’s red gum (*E*. *blakelyi*) (ACT Government 2004a). Together with white box (*E*. *albens*), these woodlands once covered a vast area of south-eastern Australia prior to European settlement [19, 20]. However, they have been profoundly affected by clearing and modification, particularly because they occurred on soils favoured for agriculture [19–22]. As a result, 92% of box-gum grassy woodlands have been cleared (over 5 million hectares), and very little of the remaining area is in good condition [20, 23], with few, if any providing ‘benchmarks’ or reference conditions to guide ecological restoration [24]. Consequently, box-gum grassy woodlands are listed nationally as a critically endangered ecological community [20, 25, 26].

A key effect of the process of modification of woodlands by humans is stand structural ‘simplification’ (Laven and Mac Nally 1998). This is a result of: (1) removal of woody debris for firewood, ‘tidying up’ and prescribed burns to manage fuel loads, (2) removal and prevention of regeneration of shrubs and trees, (3) clearing and thinning of trees [19–22, 27]. Simplification affects the value of ecosystems as habitat for biodiversity, and impacts the ecological processes that structure the ecosystem, such as nutrient cycling, fire regimes, and the spatial pattern and intensity of grazing by vertebrate herbivores.

To restore highly modified or destroyed woodlands requires an understanding of current woodland structure and composition and relationships between key elements. Through better understanding of current, simplified, woodland states we can plan and implement interventions that aim to reverse this process [28, 29].

In this study, we examined relationships between measured vegetation structure and woody debris loads in box-gum grassy woodlands in the northern part of the Australian Capital Territory (ACT) in two adjacent reserves with different management histories. Our aim was to answer three questions:

1. What are the structural and compositional characteristics of woodlands, and how do proportions of dominant species vary?
2. What are the predicted volumes of woody debris across vegetation density classes?
3. What is the relationship between woodland stand basal area and woody debris loads?

We make recommendations for future research and conservation in box-gum grassy woodlands specifically, and that also have broader relevance to woodlands generally.

## Material and methods

### Research site

**Fig 1:**
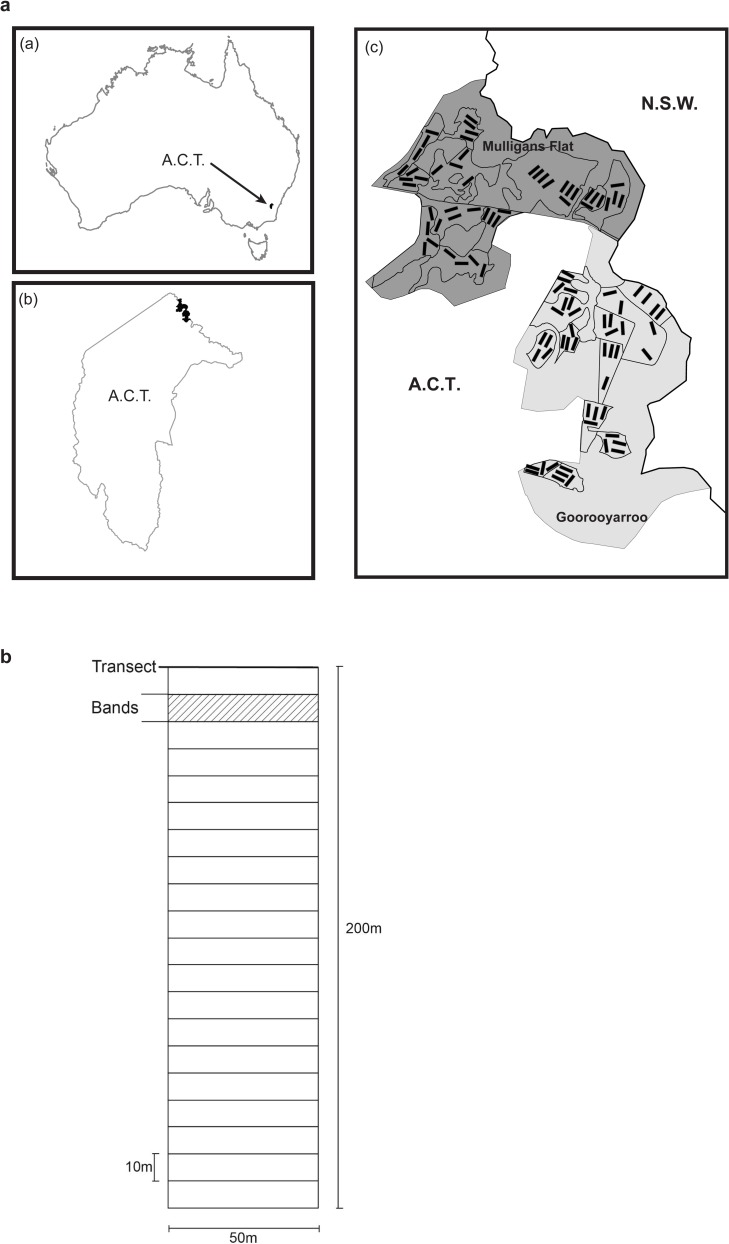
Fig 1A –The location of Mulligans Flat and Goorooyarroo Nature Reserves in the northern Australian Capital Territory (A.C.T). (a) the A.C.T within Australia. (b) the reserves within the A.C.T. (c) the experimental 1 hectare sites within the reserves. Fig 1B. A 1ha experimental site showing the method of sampling for woody debris and trees and shrubs. Woody debris was sampled using the line intersect method, with 21 x 50 metre ‘transects’ every 10 metres, measured from the eastern-most end. Trees and shrubs were measured in the 20 x 10 metre-wide ‘bands’ between transects.

The study area was in north-eastern ACT, and comprised two adjacent nature reserves—Mulligans Flat and Goorooyarroo Nature Reserves (see Fig 1A). Together, the reserves total 1494 ha, and contain 1210 ha of box-gum grassy woodland [28]. These reserves constitute the largest and most intact example of this type of woodland in the ACT [26]. Mulligans Flat Nature Reserve was reserved in 1994 [28, 29] and grazing with domestic stock and firewood collection ceased then. Neighbouring Goorooyarroo Nature Reserve was added to the nature conservation estate in 2006 and grazing with domestic stock stopped around at that time [28, 29]. A detailed site description can be found in [28] and [30].

The reserves are the location of a major designed ecological restoration experiment, “The Mulligans Flat–Goorooyarroo Woodland Experiment” (www.mfgowoodlandexperiment.org.au) [28, 29]. The aim of the experiment is to examine ways of improving box-gum grassy woodlands for biodiversity. Biodiversity being monitored includes: plants, fungi, birds, reptiles, small mammals, invertebrates and soil microbes. The inventories of woody vegetation and woody debris that we collected for this study constitute baseline data for the overall experiment. This research was conducted under ACT Government scientific licences LT2005201 and LT2009347.

### Experimental design

Our multi-level experiment consists of 24 polygons, with four 1 hectare sites per polygon (50 m x 200 m, 96 sites in total; see Fig 1A and 1B) [28]. The polygon is the key stratifying unit of this experiment. These are defined as homogenous areas of vegetation structure and type (surveyed, assessed and classified by ACT Government staff). Four combinations of broader vegetation classes describing broad structure were derived from this database:

1. High tree cover, high shrub cover (HTHS)
2. High tree cover, low shrub cover (HTLS)
3. Low tree cover, low shrub cover (LTLS)
4. Low tree cover, high shrub cover (LTHS)

These classifications describe the vegetation structure (i.e. density) of each polygon, with ‘High Tree’ and ‘High Shrub’ meaning ‘dense’ structure, and ‘Low Tree’ and ‘Low Shrub’ meaning ‘open’ structure.

### Sampling protocol

Woody debris and tree inventory data were collected at each of the 96 1 ha sites (Fig 1B). Surveys began by marking out each site using 50 m measuring tapes and flags. Starting at the eastern-most end of each site, 10m intervals were marked with flags along the 200m on both sides in 4 x 50m segments (Fig 1B). All measurements were started from right hand side looking along central axis of site from starting end. A 50m tape was laid out between 10 m interval flags either side of the site.

### Woody debris measurement

All woody debris (>2cm diameter) was measured on each transect, always commencing at the same relative side of the site, moving in the same direction. Woody debris was assessed using a continuous line intercept procedure [31, 32], recording the location on the transect and the diameter (using calipers or a diameter tape). This provided data on the number of pieces, size and spatial distribution of woody debris. At the point of intersection with the tape, the diameter of woody debris at a right angle to the central longitudinal axis was measured. Where the diameter at the point of intersection could not be measured directly (because the log was touching the ground), the nearest accessible point was measured if it has a similar diameter. Pieces of wood were tallied twice if the central longitudinal axis was intersected twice. This included forked trees and branches and trunks of the same tree. However, logs were not tallied if the central longitudinal axis ran along the line of inter-section and was not crossed [33]. Twenty one transects were assessed, including one transect at zero metres and one transect at 200 metres (21 x 50 metre transects = 1050 metres per site; see Fig 1B)

Woody debris volume was calculated using the line intercept method [31, 32] by applying the formula:

$$V=\frac{\pi^{2}\Sigma d^{2}}{8L}$$

where d is the stem diameter and L is the transect length (50 m).

### Woody vegetation

Woody vegetation was measured within the 10 m wide ‘bands between the tape transects used for woody debris measurement (20 segments per site, see Fig 1B). Where a tree had multiple stems at breast height, each stem was measured, a basal area calculated and these values were then summed.

**Fig 2:**
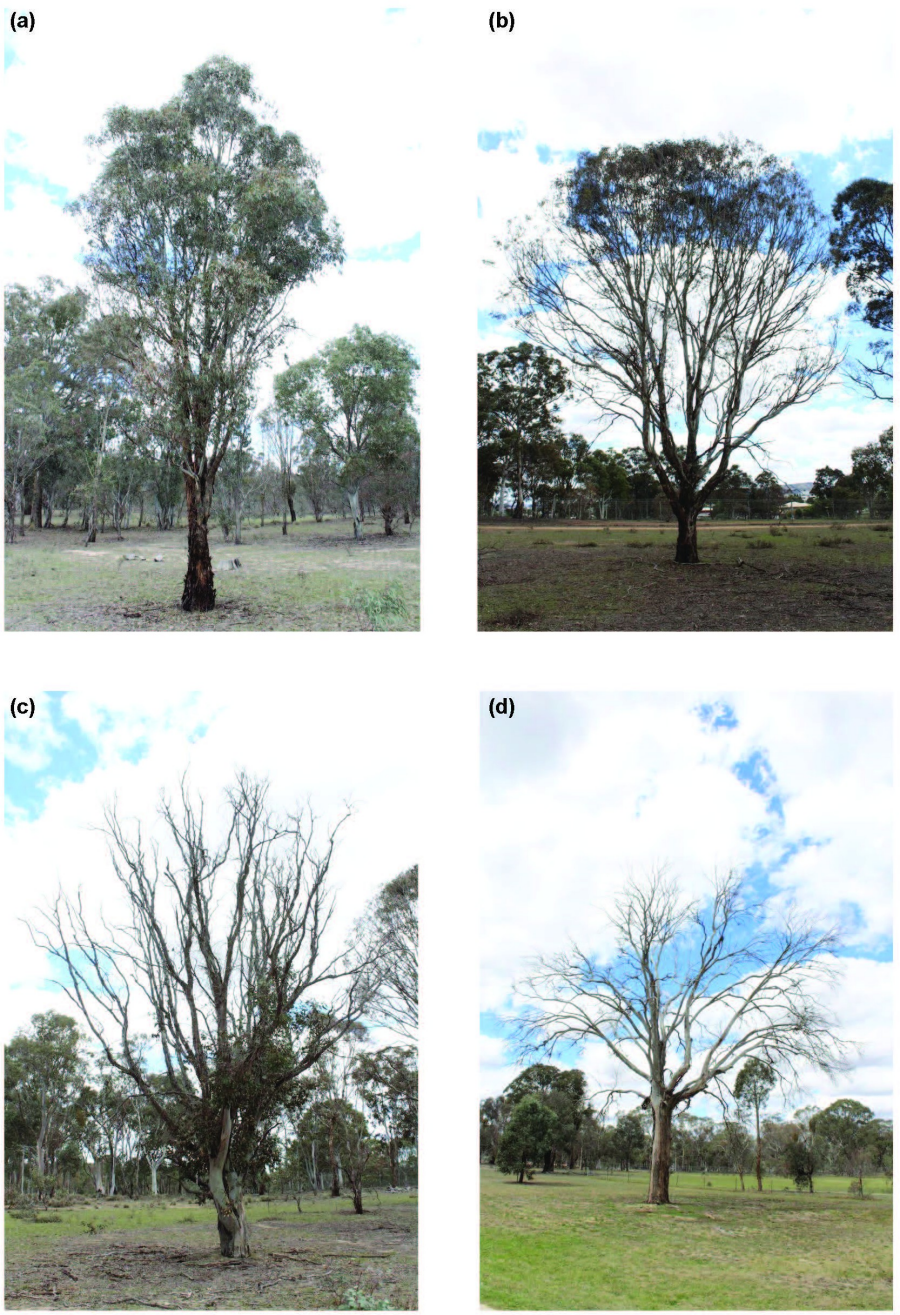
Fig 2A –An example of a tree in ‘good’ condition—full green crown, majority of leaves, twigs and branches alive and healthy. Fig 2B An example of a tree in ‘moderate’ condition—generally green crown, some leaves, twigs and branches dead. Fig 2C An example of a tree in ‘poor’ condition—sparse crown, majority of leaves, twigs and branches dead or unhealthy. Fig 2D An example of a ‘dead’ tree.

All stems over 2 cm DBH were measured. A GPS position for each tree over 10cm DBH was taken, species noted and health assessed. Health i.e. level of dieback (the phenomenon of chronic defoliation and premature death of native trees caused by a complex of biotic and abiotic factors; [34, 35], was assessed as follows:

1. good condition–full green crown, majority of leaves, twigs and branches alive and healthy (Fig 2A);
2. moderate condition—generally green crown, some leaves, twigs and branches dead (Fig 2B);
3. unhealthy; poor condition—sparse crown, majority of leaves, twigs and branches dead or unhealthy (Fig 2C);
4. dead (Fig 2D).

[31, 32]

### Statistical analysis

The data structure arising from the design was multi-level with the three levels (and hence experimental units) being reserve, polygon and site. An appropriate statistical modelling framework for assessing the effects of ‘reserve’, ‘vegetation’ and their interaction, and other relationships, was a general linear mixed model with random effects for polygon and site (see [36].

Fixed effects were estimated by least squares and random effects by restricted maximum likelihood. Statistical significance of fixed effects was assessed by Wald statistics with an appropriate adjustment for degrees of freedom [37].

## Results

### What are the structural and compositional characteristics of woodlands, and how do proportions of dominant species vary?

**Fig 3:**
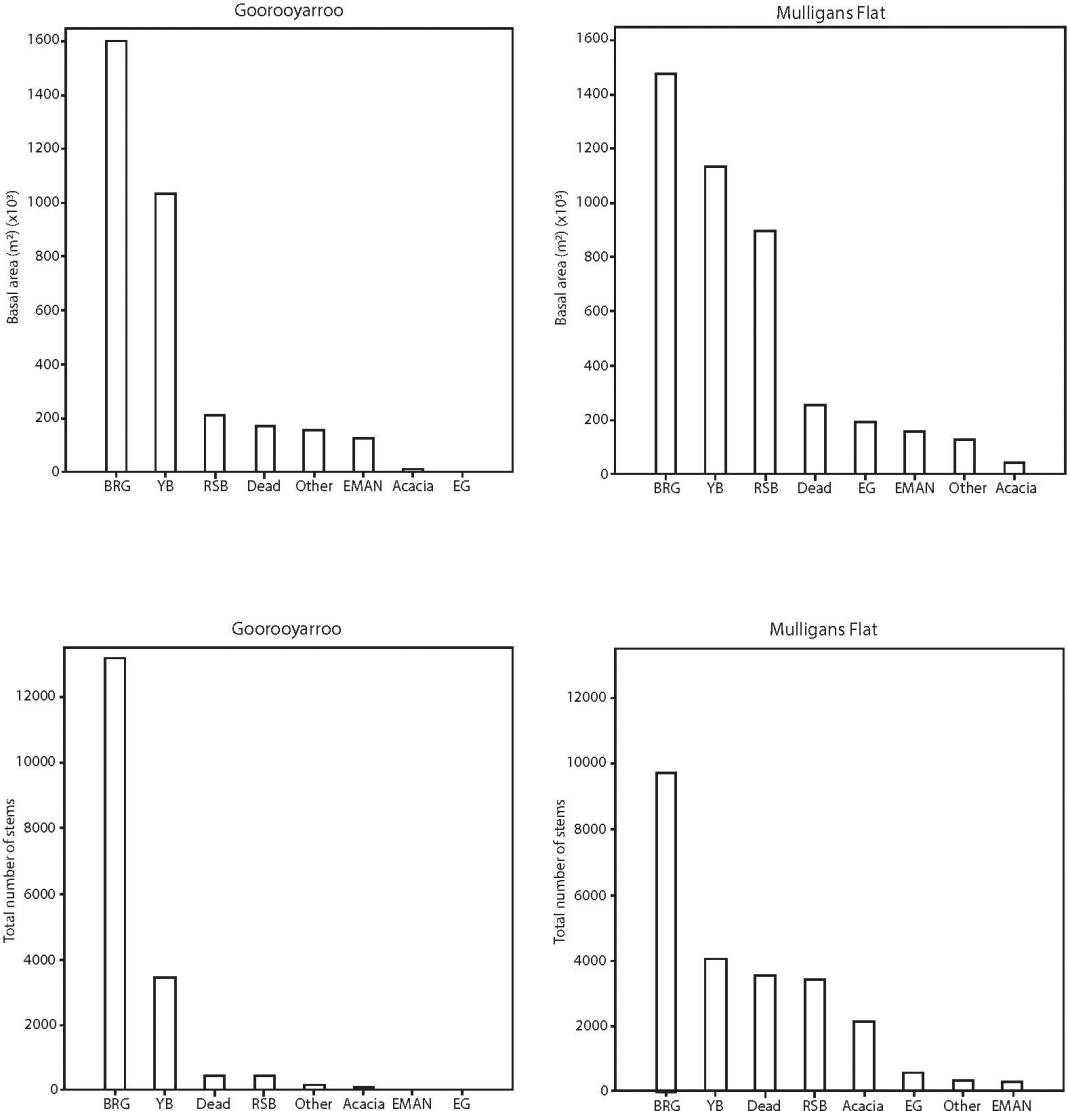
Bar-charts of basal area and number of stems for each species group by reserve.

Both Mulligans Flat and Goorooyarroo had similar tree species composition, but abundances and basal areas of trees varied (Fig 3). The two dominant species across both reserves were yellow box and *Blakely’s red gum*; although *Eucalyptus macrorhyncha* was also abundant in Mulligans Flat, but not Goorooyarroo. Blakely’s red gum was considerably less healthy than yellow box in both reserves, with around 2% of stems in good condition, 15% in moderate condition and around 80% in poor condition. In contrast, 40–50% of yellow box stems were in good condition, 40% in moderate condition, and only 10–15% in poor condition.

**Fig 4:**
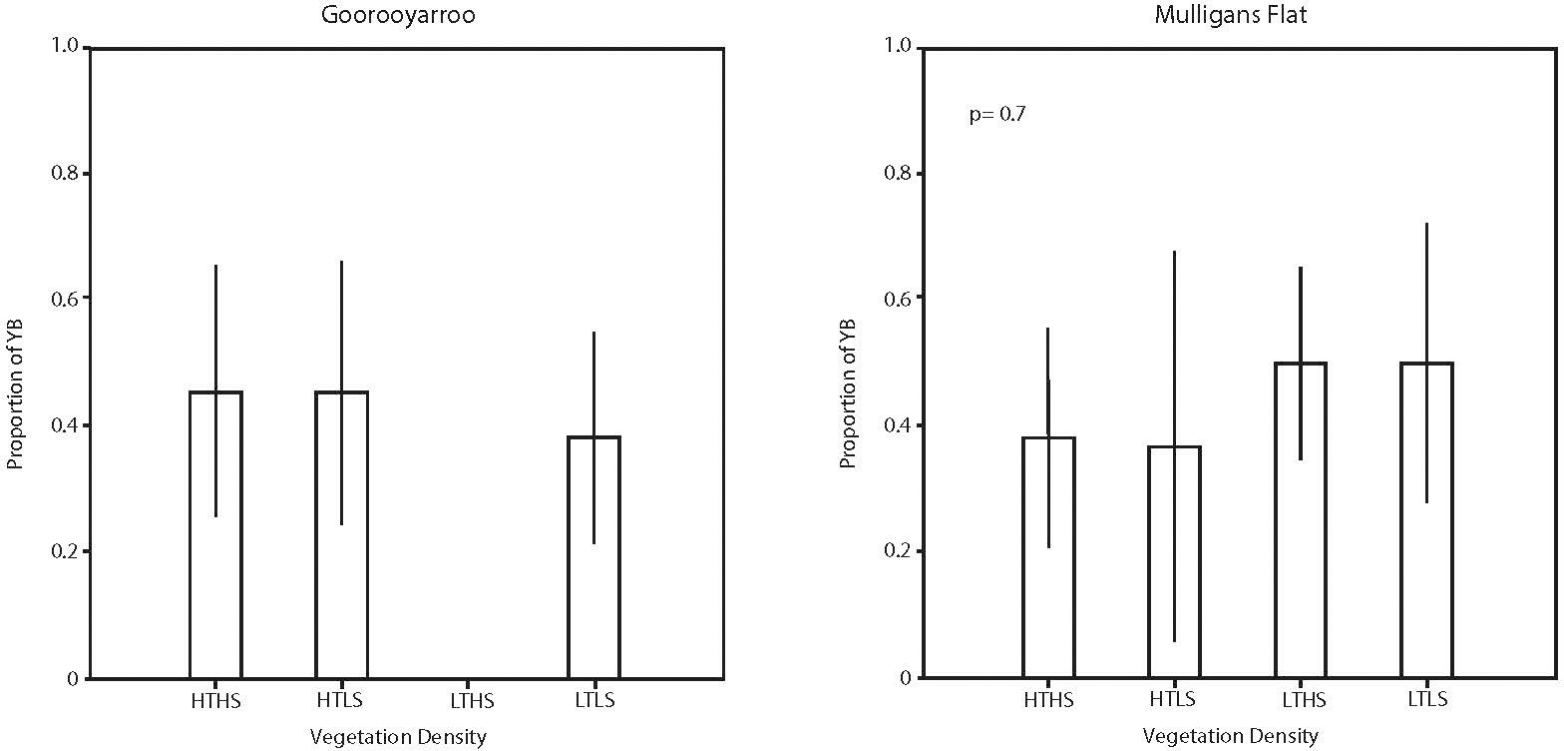
Estimated mean values and associated 95% confidence intervals for the basal area of yellow box expressed as a proportion of the basal area of yellow box and Blakely’s red gum for each vegetation class in each reserve.

**Fig 5:**
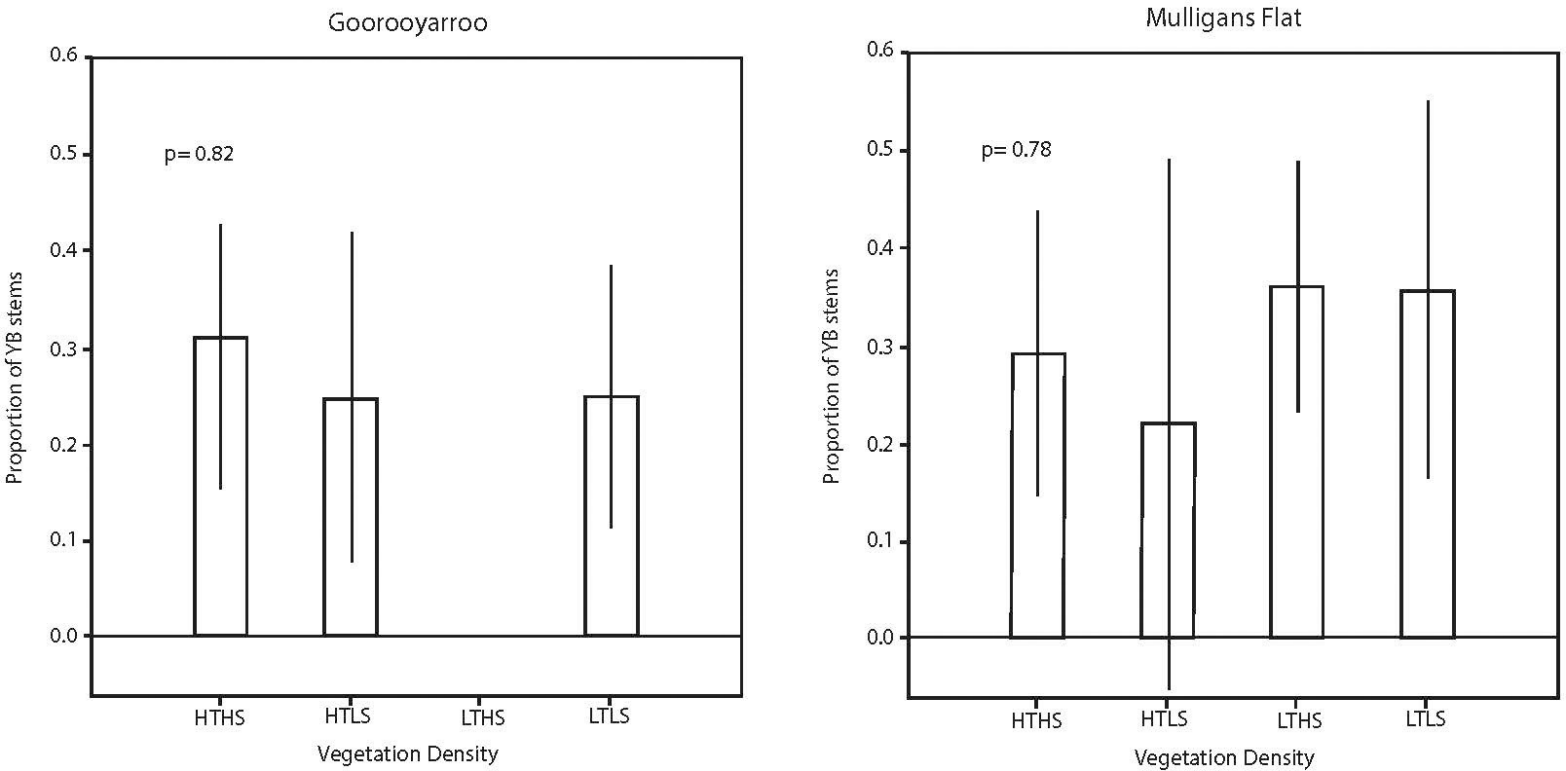
Estimated mean values and associated 95% confidence intervals for the number of stems of yellow box expressed as a proportion of the number of stems of yellow box and Blakely’s red gum for each vegetation class in each reserve.

The basal area of yellow box was expressed as a proportion of the basal area of yellow box and Blakely’s red gum. There was no evidence of a difference in the proportion of yellow box basal area associated with reserve or vegetation class (Fig 4). The number of stems of yellow box was expressed as a proportion of the number of stems of yellow box and Blakely’s red gum. There was no evidence of a difference in the proportion of the number of stems of yellow box within each reserve or by vegetation class (Fig 5).

In summary, the ratio of basal area (approximately 50:50) and number of stems (between 25:75 and 30:80) for yellow box versus Blakely’s red gum, the proportion did not change significantly with reserve or vegetation classification.

### What are the predicted volumes of woody debris across vegetation density classes?

**Fig 6:**
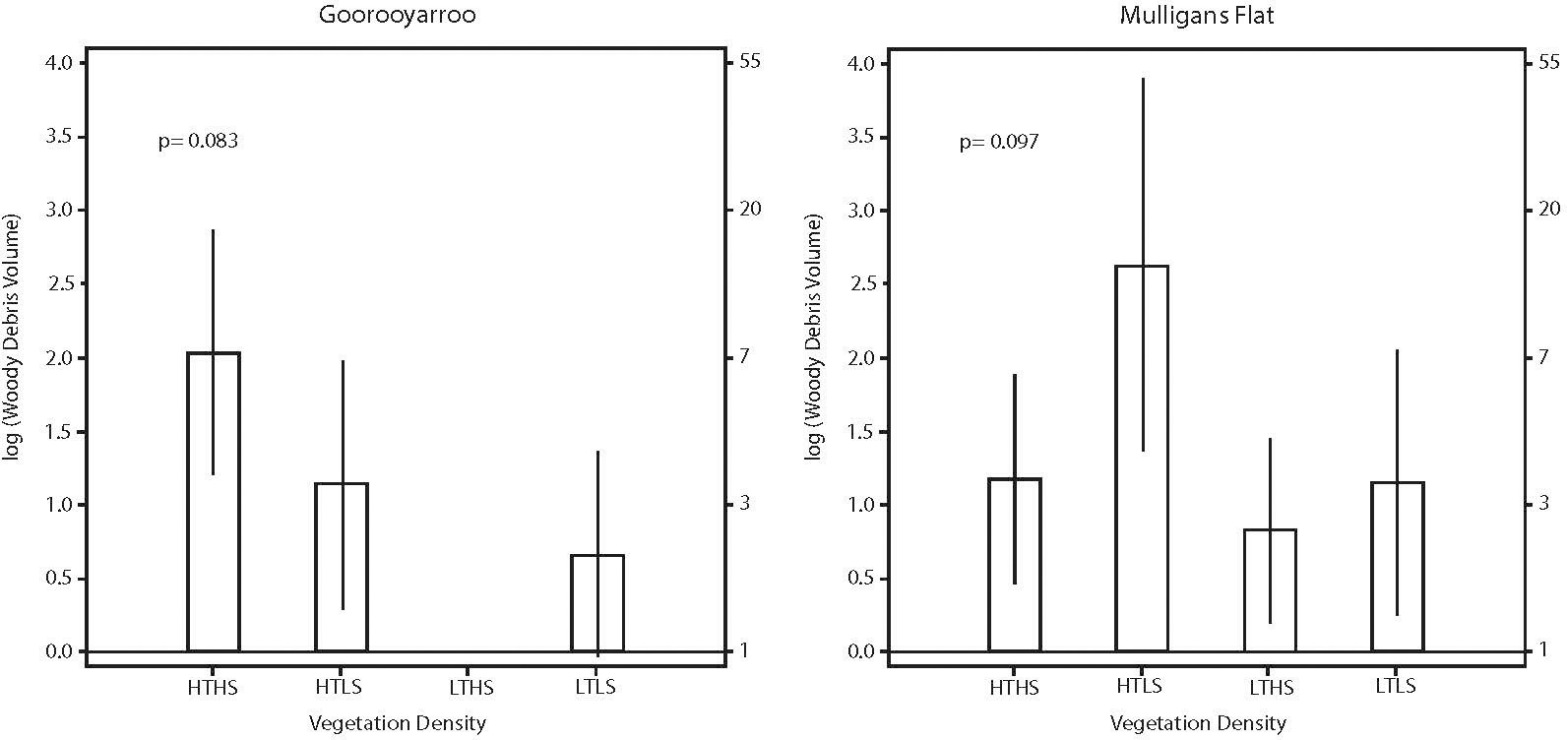
Predicted woody debris loads across vegetation classes. Estimated mean values and associated 95% confidence intervals for log (volume of woody debris) for each vegetation class in each reserve.

Predicted volume of woody debris (derived by back-transformation) varied between reserves and vegetation class (Fig 6). The predicted mean woody debris volume (with associated confidence intervals) ranged from 13.82 m^3^ (3.88, 49.23) to 2.28 m^3^ (1.20, 4.35) in Mulligans Flat and from 7.54 m^3^ (3.31, 17.19) to 1.93 m^3^ (0.96, 3.86) in Goorooyarroo.

### What is the relationship between woodland stand basal area and woody debris loads?

**Fig 7:**
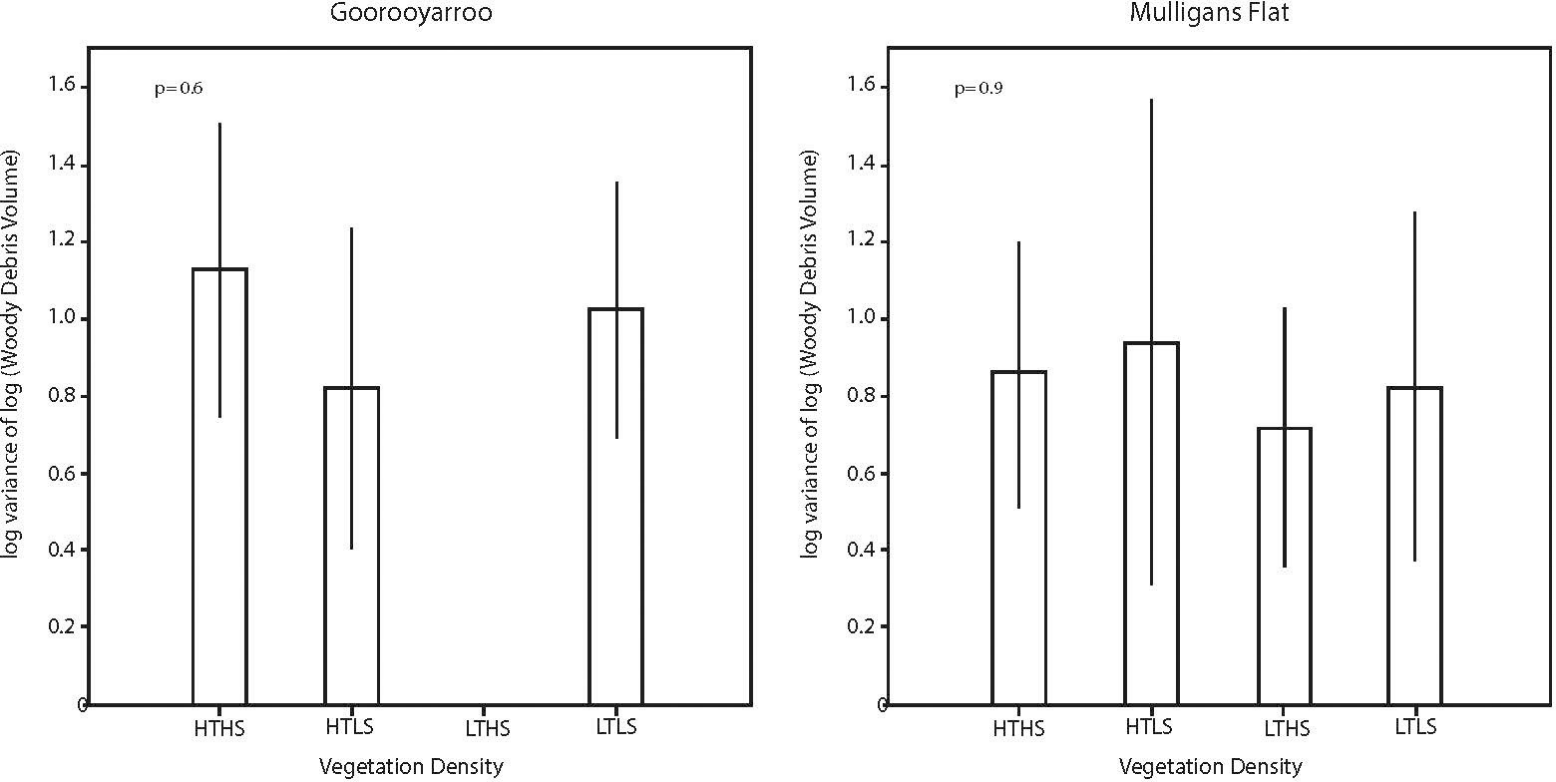
Heterogeneity of woody debris volume. Estimated mean values and associated 95% confidence intervals for log(variance of woody debris volume) for each vegetation class in each reserve.

There was no evidence of any variation in heterogeneity of pattern of woody debris loads within sites (i.e. between bands–Fig 1B) across vegetation class within each reserve. This suggests that woody debris deposition pattern within sites was not influenced by reserve or vegetation class (Fig 7), and therefore is essentially ‘natural’ in distribution.

**Fig 8:**
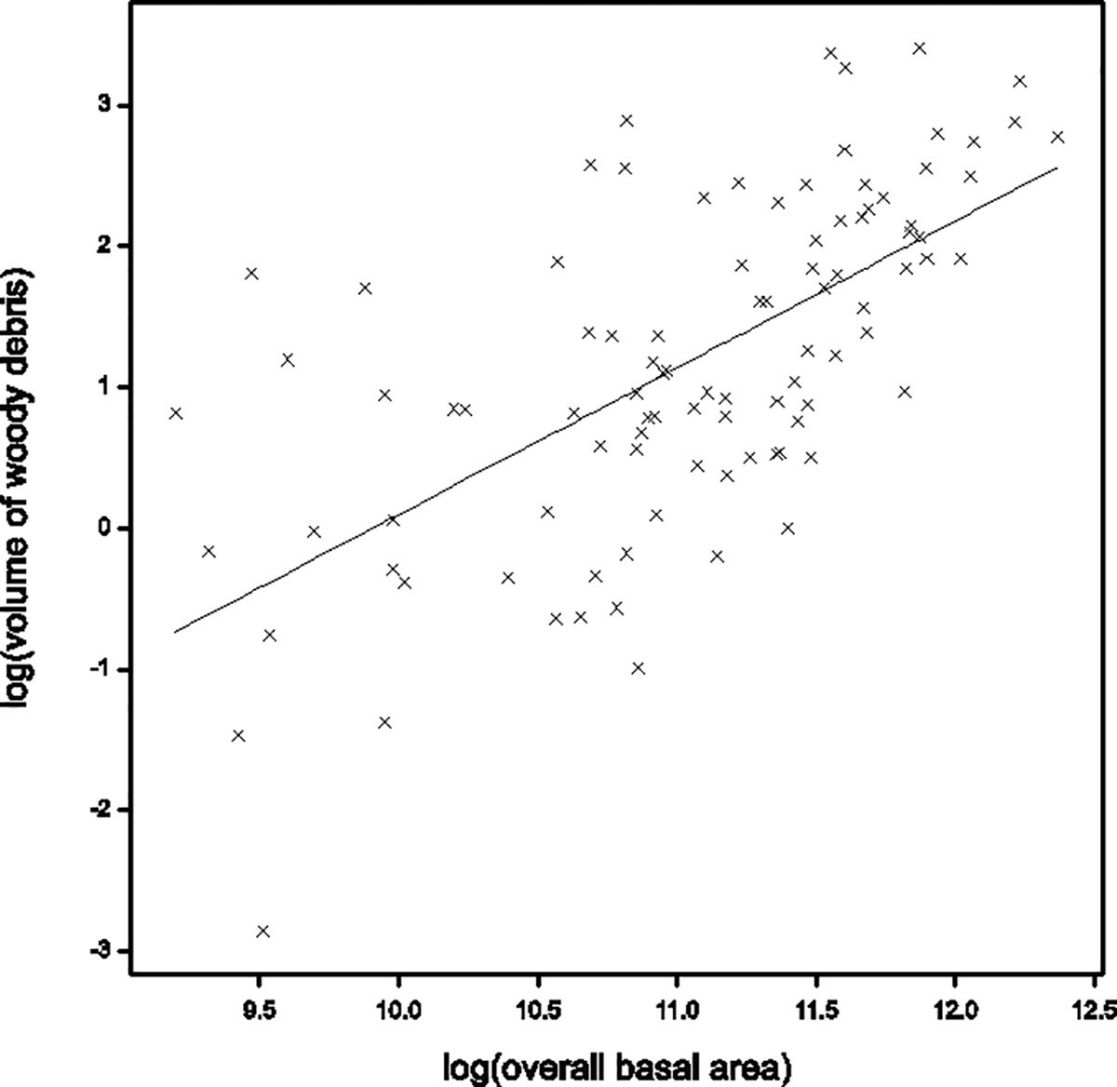
The relationship between log(woody debris volume) and log(overall basal area) was strong and highly significant (p<0.001, slope 1.038 se 0.1392). This means there was a direct 1:1 relationship between the two measures.

Fine woody debris (2–10 cm diameter) and coarse woody debris (>10 cm diameter) were analysed separately, but the result was similar to that for both combined (termed “woody debris”). There was a strong and highly significant (p<0.001, slope 1.038 se 0.1392; Fig 8) relationship between log woody debris volume and log basal area overall. There was no evidence that the relationship differed between reserve and/or vegetation class. The relationship was a direct 1:1 relationship between log(woody debris volume) and log(overall basal area) i.e.woody debris α basal area. Thus, for a 1% increase in basal area, woody debris will increase by 1%. For example, if basal area increased from 5.99 m^2^ to 6.05 m^2^ (a 1% increase), woody debris would be predicted to increase from 3.11 m^3^ to 3.15 m^3^ (a 1% increase) tonnes per ha. Although this relationship is strong and statistically significant, prediction of woody debris at specific levels of basal area is imprecise due to the high variability around the relationship between the two variables.

## Discussion

### What are the structural and compositional characteristics of woodlands, and how do proportions of dominant species vary?

There were two dominant tree species within the experimental woodlands: yellow box and Blakely’s red gum. The different contributions that the two dominant tree species made are important because of the different habitat structures (e.g. litter type and bark roughness) and species associations (e.g. beetles) that each species supports. In this study, the proportions of yellow box basal area and stems to Blakely's red gum was the same in both reserves and across all vegetation classes. This is important information, as each species contributes different ecological values to the woodland environment. For example, McElhinny et al. (2010) found that the amount of litter input from yellow box and Blakely's red gum was not significantly different. However, they did find that Blakely's red gum produced significantly higher loads of leaf and other kinds of litter whereas yellow box had significantly more fine twig litter. These litter differences affect beetle assemblages and functional groups, and a proportion of the beetle assemblages associated with both species are mutually exclusive [38]. Yellow box is generally a rough-barked tree and Blakely's red gum is generally smooth barked [39]. Rough-barked trees provide important habitat for species typical of box-gum grassy woodlands, such as the white-throated treecreeper *Cormobates leucophaea* [40]. Large Blakely's red gum are favoured nest trees for the superb parrot *Polytelis swainsonii* [41] and provide important habitat in landscapes where it is found [42]. Therefore, maintaining or managing for the proportions of the two species in woodlands is important for biodiversity.

Blakely's red gum had a larger basal area and a greater number of stems than any other species. This supports the observation that the species can regenerate vigorously given suitable conditions. Stands of yellow box, the next most abundant tree species in our area, were characterised by considerably fewer stems, but a similar basal area because individuals of the species had a larger mean DBH. Yellow box also was considerably healthier (i.e. less dieback) than Blakely's red gum. These factors, and the consistent proportion of the two species across reserves and vegetation classes (Figs 4 and 5; see below), suggest that the life strategies differ between the two species (i.e. Blakely’s red gum producing many stems that self-thin versus yellow box producing fewer stems that are more healthy). There have been some concerns that the cessation of grazing of woodlands, and the effects of climate change, could result in vegetation thickening of woodlands [43, 44], which may have effects on biodiversity and ecological processes. It will be important to track the changes in the yellow box and Blakely's red gum populations over time (and examine effects on biodiversity and ecological processes) to examine whether vegetation thickening occurs in the long-term, and what the ecological consequences are.

### What are the predicted volumes of woody debris across vegetation density classes?

This study adds to the growing body of work on woody debris loads in box-gum grassy and similar woodlands [2, 45, 46]. As found in [47], there was no significant difference in woody debris volumes between the two reserves (Fig 6) or the heterogeneity of that woody debris (Fig 7). Predicted mean volumes of woody debris for the reserves were lower (3.43 m^3^ per ha for Goorooyarroo and 3.14 m^3^ per ha for Mulligans Flat) than those published for box-gum grassy woodlands elsewhere [24, 48]. There was no significant difference in predicted volumes between the reserves. Gibbons et al. (2008) predicted 8.2 m^3^ per ha (+/- 2.6) for yellow box communities, and Killey et al. (2010) predicted 7.0 m^3^ per ha (based on tree level analysis). The estimates in this study are also less than those estimated CWD volumes in [47](3.0 m^3^ to 247 m^3^ per ha, mean 34 m^3^ for both reserves). The relatively low volumes found in these woodlands reflects the DBH (i.e. age) profile of the trees. Killey et al. (2010) studied yellow box in Goorooyarroo and found that yellow box trees that were 100cm DBH were 10 times more likely to produce CWD than a 50cm DBH tree, and produced 10 times more CWD volume. Most trees measured in this study were under 40 cm DBH, with few over 50 cm DBH. So while this DBH profile indicates a woodland ecosystem in recovery (i.e. many young trees), and the heterogeneity of the woody debris (see below) indicate ‘natural’ falls, the low volumes highlight the slow rate of accumulation of this critical resource. as part of the recovery process. This is an issue that potentially acts as a barrier to persistence of biodiversity and its recovery [9].

While mean woody debris volumes in the reserves were relatively uniform, these figures mask some important information about patterns of woody debris in relation to structure. Heterogeneity of woody debris can affect biodiversity [49, 50]. We found high variability in woody debris volume across all sites (Fig 7), and in relation to vegetation structure. These results highlight the importance of both volume and variability of pattern (i.e. heterogeneity) of woody debris.

Our study gives managers an indication of the expected volumes for different woodland structures, and also goals for volumes of woody debris to manage for where it is lacking (e.g. highly modified paddocks with remnant scattered trees). Results also indicate the level of ‘natural’ variability in woody debris patterns, and indicate that this should be replicated if woody debris is being added to a site.

### What is the relationship between woodland stand basal area and woody debris loads?

There was a strong 1:1 relationship between overall basal area of a site and woody debris volumes. This means that managers of areas of box-gum grassy woodlands from which woody debris has been removed can use the basal area of a site to determine what the minimum woody debris volume should be. An example of such a situation could be where a paddock with only scattered trees is to be restored to functional box-gum woodland. Logically, if there are more, larger trees per ha, basal area can be maintained in a woodland but that woodland can be open (i.e. fewer stems) and still produce the same amount of woody debris and litter. For example, 5.99 m^2^ basal area per hectare (see above) could constitute 7.6 trees at 100 cm DBH, or 30.5 trees at 50 cm DBH.

Prior to European settlement, there would have been more, large eucalypts in box-gum grassy woodlands [51]. More, larger trees would have produced proportionally more woody debris [52]. It is therefore probable that that woody debris loads in pre-European woodlands were much higher than those estimated in this study or those measured in studies of benchmark sites [24]. Therefore, 1:1 should be the minimum volume because evidence suggests that greater volumes can support higher levels of biodiversity [9, 50].

In light of our findings, management of box-gum grassy woodlands should consider the following:

1. aim for approximately a 50:50 ratio of yellow box to Blakely's red gum basal area in woodland restoration projects;
2. add deadwood to accelerate the recovery of woodland structure, and should be, at a minimum, added proportionally 1:1 to standing basal area (i.e. a basal area of 5.99 m^2^ requires a minimum volume of 3.11 m^3^), and higher volumes if possible to enhance biodiversity [9, 50].
3. manage for both volume and heterogeneity of woody debris loads;
4. retain large trees to (a) harness proportionally higher woody debris and litter inputs, (b) create more open woodland while maintaining these inputs. This could be achieved by encouraging tree regeneration or planting in woodlands with low tree and low shrub cover and/or maintaining woodland structural and compositional heterogeneity i.e. have a range of all four broad vegetation classifications.

## Conclusion

The field sampling approach and resulting data collected for this study provide the basis for tracking the effects of differing woodland structures and composition on biodiversity and ecological processes. In future, it will be important to model how tree populations change through time, and in relation to existing vegetation structure and experimental treatments, and how this affects biodiversity and ecosystem processes. The relative recruitment and survival of small stems of yellow box and Blakely's red gum, and the associated cascading ecosystem effects on biodiversity will be important information for managers in deciding whether or not to intervene with management actions. Although the characteristics such as woody debris volume and heterogeneity within these reserves suggest a ‘natural’ or undisturbed woodland, the paucity of larger trees reflecting a recovering ecosystem rather than a ‘pristine’ benchmark site. The recovery of key structures in these box-gum grassy woodlands has probably been taking place for a longer period than the formal designation of the reserves. Our results highlight the slow accumulation of woody debris, even after vegetation recovery has been underway for some time. This supports the argument for the addition of woody debris in such woodlands in Australia, and also may have applicability to other woodlands elsewhere.
